# Alemtuzumab‐induced petechiae and epistaxis in a patient with relapsing–remitting multiple sclerosis: A case report

**DOI:** 10.1002/ccr3.8143

**Published:** 2023-11-25

**Authors:** Farhad Mahmoudi, Sayed Ali Emami, Farid Masaeli, Najmeh Rayatpisheh

**Affiliations:** ^1^ School of Medicine Isfahan University of Medical Sciences Isfahan Iran; ^2^ School of Medicine Shahrekord University of Medical Sciences Shahrekord Iran

**Keywords:** alemtuzumab, DMT, epistaxis, immune thromboctopenic purpura, ITP, MS, multiple sclerosis, RRMS

## Abstract

Here, we present a case of relapsing–remitting multiple sclerosis that experienced petechiae and epistaxis following treatment with second dose of alemtuzumab. This study highlights such effects, emphasizing the need for vigilance as alemtuzumab usage increases. Timely recognition and management are vital for patient care.

## Graphical abstract

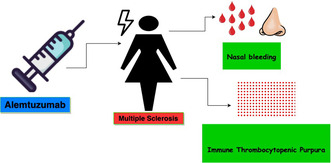

## 1 INTRODUCTION

Multiple sclerosis (MS) is an autoimmune disease that affects the central nervous system. The course of the disease varies among individuals, leading to a wide range of symptoms and patterns of presentation. Additionally, there are other conditions, such as radiologically isolated syndrome, which can progress to MS.^1^ While there is no curative therapy for this condition, certain drugs can modify the course of the disease and improve the prognosis and quality of life for patients. Disease‐modifying treatments (DMTs) are the preferred treatment option for patients with MS. Alemtuzumab is an intravenously administered DMT drug that has been available since 2014 for the treatment of patients with MS who have not responded adequately to two or more DMTs. It is a recombinant DNA‐derived humanized monoclonal antibody that selectively binds to the CD52 antigen on B and T lymphocytes, depleting them from the bloodstream. This drug has been described as a safe and effective treatment with minimal side effects for patients with relapsing–remitting multiple sclerosis.^2^ Infusion‐associated reactions (IARs) was the most frequent adverse effects during treatment with alemtuzumab. The reactions generally included headache, rash, fever, nausea, flushing, urticaria, insomnia, and itching. It is thought that the IARs are due to allergic reactions of hypersensitivity mediated by immunoglobulin E (IgE) and nonallergic cytokine release reactions induced by the recruitment of inflammatory cells or cell lysis.^3^

While there are few studies on the cutaneous adverse effects of alemtuzumab, in this study, we report the first case of an MS patient treated with alemtuzumab who developed drug‐induced petechiae and epistaxis. Physicians should be capable of recognizing and understanding this adverse reaction pattern to improve clinical management and inform patients about potential side effects.

## 2 CASE PRESENTATION

A 58‐year‐old Caucasian woman with relapsing–remitting multiple sclerosis for 32 years, who had no previous medical conditions, presented to the emergency department due to muscle spasms and stiffness in her right foot. Neurological examination revealed weakness in her right lower limb with 3/5 muscle strength. She was diagnosed with MS at the age of 26 through a magnetic resonance imaging (MRI) and had previously been on weekly 30 mcg interferon beta‐1a (IFNb) treatment. During acute MS attacks, she received intravenous corticosteroids. Despite being on disease‐modifying therapies (DMTs), she experienced more relapses in the last year, and her Expanded Disability Status Scale (EDSS) increased from 2 to 3. During the current hospitalization, the patient underwent brain and cervical spinal MRI, which revealed the appearance of new periventricular white active plaques in addition to previous black old lesions.

Considering the progression of her disease condition, she was started on treatment with alemtuzumab at a dose of 12 mg/day IV. After receiving the first dose of alemtuzumab, the patient did not experience any drug reactions. Following the completion of the treatment, her muscle spasms and stiffness resolved, and she was discharged from the hospital.

The day after, the patient came to the hospital to receive the second dose of the drug. While receiving the treatment, her condition remained stable, and she did not experience any notable symptoms. However, 1 day after finishing the second dose of alemtuzumab, the patient started to experience a gradual appearance of petechiae on her upper and lower limbs, chest, shoulders, and back. Moreover, a few minutes later, she developed epistaxis. Her blood pressure and heart rate were 127/73 mm Hg and 87 BPM, respectively. The patient denied any previous history of eczema, skin disease, or respiratory allergies.

A complete blood count revealed a hemoglobin (Hb) level of 13.8 g/dL, a white blood count (WBC) of 7100/μL, a mean cell volume (MCV) of 86 fl, and a platelet count (PLT) of 89 × 10^3/μL. Bleeding time (BT), prothrombin time (PT), and partial thromboplastin time (PTT) were all within the normal range. The patient did not exhibit any clinical indicators of thrombotic thrombocytopenic purpura (TTP), hemolytic uremic syndrome (HUS), or disseminated intravascular coagulation (DIC). Additionally, laboratory tests yielded negative results for these conditions.

**FIGURE 1:**
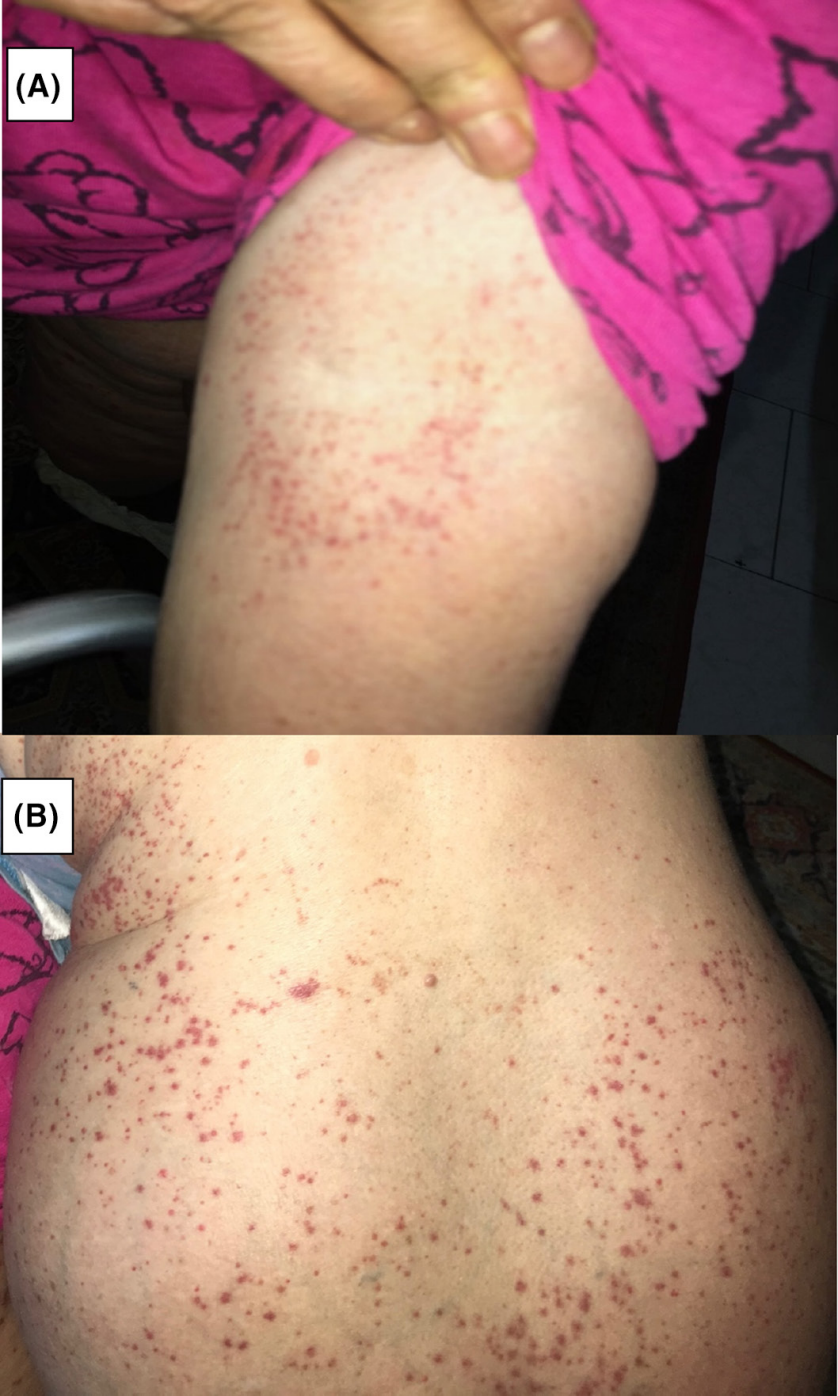
Cutaneous drug reaction lesions. Petechiae in a 58‐year‐old Caucasian woman on left antecubital (A) and lower back (B) 1 day after receiving the second dose of alemtuzumab.

Dermatology was consulted to investigate the possible causes of petechiae, and ENT was consulted to manage and evaluate the epistaxis. Despite applying pressure to the nostrils and placing ice on the forehead, the epistaxis did not cease. Consequently, the patient underwent posterior nasal packing, which successfully stopped the bleeding (Figure 1).

## 3 DISCUSSION

RRMS is the most common form of MS, including approximately 85% of MS cases. In RRMS, distinct neurological symptoms arise from inflammatory attacks within the central nervous system (CNS) known as “relapses.” These relapses are followed by periods of either full or partial recovery from both new and preexisting symptoms, referred to as “remissions.” Initiating DMTs in the early stages of the disease to stop the immune attack on the CNS has the potential to enhance long‐term clinical outcomes by reducing the accumulation of neurological damage that may occur during the initial phases of the disease. Effectively managing relapses is crucial to prevent the worsening of disability in individuals with RRMS. Currently, there are more than 18 distinct DMT options, each with unique mechanisms of action, approved by the US Food and Drug Administration (FDA) for the treatment of RRMS. The selection of the most appropriate DMT for newly diagnosed MS patients is a topic of significant debate in the field of MS care.^4^ Alemtuzumab is known as one of the medications used in patients with relapsing–remitting multiple sclerosis who have not responded to other drugs. This medication has some side effects, such as headache, rash, itching, fever, fatigue, hypothyroidism, lymphopenia, redness of the face and neck, and more.^5^

There are many underlying causes that can lead to the development of generalized petechiae, including prolonged straining, infectious diseases, and adverse effects of medications. Adverse cutaneous reactions to drugs typically begin within 12–24 h after exposure.^6^ The exact mechanism behind this thrombocytopenia is not entirely clear. Nevertheless, there have been several theories proposed to elucidate the mechanism of ITP in individuals treated with alemtuzumab. One hypothesis suggests that alemtuzumab induces the selective depletion and predominant proliferation of T cells, stimulated by elevated serum levels of interleukin‐21 (IL‐21). Another theory suggests that autoimmunity may arise from the rapid repopulation of B‐lymphocytes, a phenomenon primarily observed in individuals with a genetic predisposition for autoimmune conditions. Both of these hypotheses center on the concept of abnormalities during lymphocyte reconstitution.^7^

Cuker et al. reported a case series of six patients with MS treated with alemtuzumab, resulting in the development of immune thrombocytopenia (ITP). Five of these patients achieved complete remission after treatment, while one unfortunately succumbed to the condition, underscoring the need for serious consideration of this adverse effect.^8^

Similarly, there are several reasons for epistaxis, such as nose picking, dry air, allergies, sinusitis, trauma to the nose, chronic alcohol use, bleeding disorders like hemophilia, cocaine use, and medication side effects. Laboratory tests, past medical history, family history, and a physical examination of the patient ruled out all of these causes, except for the adverse effects of the medication. The patient's symptoms manifested 1 day after receiving the second dose of alemtuzumab, making a drug reaction a highly plausible explanation for these symptoms. Although rare, to the best of our knowledge, this is the first case report of generalized petechiae and epistaxis following treatment with alemtuzumab, which was completely resolved after treatment. This case report suggests that alemtuzumab may induce changes that lead to epistaxis, as nasal bleeding is not routinely observed in MS patients. In our case, ITP and epistaxis was attributed to alemtuzumab treatment, as no other underlying cause was identified. The results underscore the significance of patient education and comprehensive clinical monitoring for promptly identifying symptoms and effectively managing ITP. As soon as petechiae develop, the patient should promptly seek medical help and be admitted to the hospital for further treatment. The sooner the patient seeks help, the more effective the treatment can be, leading to reduced complications.

## 4 CONCLUSION

Generalized petechiae and epistaxis are uncommon side effects of alemtuzumab treatment in MS patients. Nonetheless, with the increasing use of alemtuzumab in MS treatment, healthcare providers should be able to identify and comprehend this adverse reaction pattern in order to enhance clinical management and provide patients with information about potential adverse effects.

Further research efforts are necessary to explore potential underlying factors in the MS patient population that could make them more vulnerable to adverse effects, like generalized petechiae and epistaxis, following alemtuzumab treatment.

## AUTHOR CONTRIBUTIONS

**Farhad Mahmoudi:** Conceptualization; data curation; investigation; methodology; resources; writing – original draft; writing – review and editing. **Sayed Ali Emami:** Writing – original draft; writing – review and editing. **Farid Masaeli:** Writing – original draft; writing – review and editing. **Najmeh Rayatpisheh:** Writing – original draft; writing – review and editing.

## FUNDING INFORMATION

No funding was received for this article.

## CONFLICT OF INTEREST STATEMENT

The authors have no conflicts of interest to declare.

## ETHICS STATEMENT

The patient has been de‐identified. Any images used do not permit the identification of the individual. Otherwise, there are no ethical concerns in this manuscript. There was no ethics approval required for this manuscript.

## CONSENT

Written informed consent was obtained from the patient to publish this report in accordance with the journal's patient consent policy and with institutional guidelines.

## WRITTEN CONSENT FOR PUBLICATION

All of the authors have provided their consent to publication.

## CODE AVAILABILITY

Not applicable.

## Data Availability

All of the data and material are available.

## References

[1] Fereidan‐Esfahani M , Mahmudi F , Jahansouz M , Weber MS , Rodriguez M . Biomarkers in radiologically isolated syndrome: the missing piece in the puzzle of treatment indication? J Neurol Sci. 2017;375:129. 28320114 10.1016/j.jns.2017.01.055

[2] Wilken J , Traboulsee A , Nelson F , et al. Longitudinal assessment of neurocognitive function in people with relapsing multiple sclerosis initiating alemtuzumab in routine clinical practice: LEM‐COG study results. Mult Scler Relat Disord. 2023;73:104677. 37028124 10.1016/j.msard.2023.104677

[3] Caon C , Namey M , Meyer C , et al. Prevention and management of infusion‐associated reactions in the comparison of alemtuzumab and Rebif(®) efficacy in multiple sclerosis (CARE‐MS) program. Int J MS Care. 2015;17(4):191‐198. 26300705 10.7224/1537-2073.2014-030 PMC4542714

[4] Freeman L , Longbrake EE , Coyle PK , Hendin B , Vollmer T . High‐efficacy therapies for treatment‐naïve individuals with relapsing‐remitting multiple sclerosis. CNS Drugs. 2022;36(12):1285‐1299. 36350491 10.1007/s40263-022-00965-7 PMC9645316

[5] Guarnera C , Bramanti P , Mazzon E . Alemtuzumab: a review of efficacy and risks in the treatment of relapsing remitting multiple sclerosis. Ther Clin Risk Manag. 2017;13:871‐879. 28761351 10.2147/TCRM.S134398 PMC5522829

[6] Tempark T , John S , Rerknimitr P , Satapornpong P , Sukasem C . Drug‐induced severe cutaneous adverse reactions: insights into clinical presentation, immunopathogenesis, diagnostic methods, treatment, and pharmacogenomics. Front Pharmacol. 2022;13:832048. 35517811 10.3389/fphar.2022.832048 PMC9065683

[7] Sarvepalli D , Rashid MU , Ullah W , Zafar Y , Khan M . Idiopathic thrombocytopenic purpura: a rare syndrome with alemtuzumab, review of monitoring protocol. Cureus. 2019;11(9):e5715. 31720183 10.7759/cureus.5715 PMC6823085

[8] Cuker A , Coles AJ , Sullivan H , et al. A distinctive form of immune thrombocytopenia in a phase 2 study of alemtuzumab for the treatment of relapsing‐remitting multiple sclerosis. Blood. 2011;118(24):6299‐6305. 21960587 10.1182/blood-2011-08-371138

